# Exploring a multiparameter MRI–based radiomics approach to predict tumor proliferation status of serous ovarian carcinoma

**DOI:** 10.1186/s13244-024-01634-7

**Published:** 2024-03-18

**Authors:** Li Liu, Ling Zhao, Yang Jing, Dan Li, Hua Linghu, Haiyan Wang, Linyi Zhou, Yuan Fang, Yongmei Li

**Affiliations:** 1 Department of Radiology, The People’s Hospital of Yubei District of Chongqing City, No. 23 ZhongyangGongyuanBei Road, Yubei District, Chongqing, 401120 China; 2 Department of Radiology, The First Affiliated Hospital of Chongqing Medical University, No. 1 Youyi Road, Yuzhong District, Chongqing, 400016 Yuanjiagang China; 3 Huiying Medical Technology Co., Ltd, Dongsheng Science and Technology Park, Room A206, B2Haidian District, Beijing, 100192 China; 4 Department of Pathology, Chongqing Medical University, No.1 Medical College Road, Yuzhong District, Chongqing, 400016 China; 5 Department of Obstetrics and Gynecology, The First Affiliated Hospital of Chongqing Medical University, No. 1 Youyi Road Yuzhong District, Chongqing, 400016 Yuanjiagang China; 6 Department of Radiology, Army Medical Center, Daping Hospital, Army Medical University, 10# Changjiangzhilu, Chongqing, 40024 China

**Keywords:** Serous ovarian carcinoma, Tumor proliferation status, Ki-67, Radiomics, MRI

## Abstract

**Objectives:**

To develop a multiparameter magnetic resonance imaging (MRI)-based radiomics approach that can accurately predict the tumor cell proliferation status of serous ovarian carcinoma (SOC).

**Materials and methods:**

A total of 134 patients with SOC who met the inclusion and exclusion criteria were retrospectively screened from institution A, spanning from January 2016 to March 2022. Additionally, an external validation set comprising 42 SOC patients from institution B was also included. The region of interest was determined by drawing each ovarian mass boundaries manually slice-by-slice on T2-weighted imaging fat-suppressed fast spin-echo (T2FSE) and T1 with contrast enhancement (T1CE) images using ITK-SNAP software. The handcrafted radiomic features were extracted, and then were selected using variance threshold algorithm, SelectKBest algorithm, and least absolute shrinkage and selection operator. The optimal radiomic scores and the clinical/radiological independent predictors were integrated as a combined model.

**Results:**

Compared with the area under the curve (AUC) values of each radiomic signature of T2FSE and T1CE, respectively, the AUC value of the radiomic signature (T1CE-T2FSE) was the highest in the training set (0.999 vs. 0.965 and 0.860). The homogeneous solid component of the ovarian mass was considered the only independent predictor of tumor cell proliferation status among the clinical/radiological variables. The AUC of the radiomic–radiological model was 0.999.

**Conclusions:**

The radiomic–radiological model combining radiomic scores and the homogeneous solid component of the ovarian mass can accurately predict tumor cell proliferation status of SOC which has high repeatability and may enable more targeted and effective treatment strategies.

**Critical relevance statement:**

The proposed radiomic–radiological model combining radiomic scores and the homogeneous solid component of the ovarian mass can predict tumor cell proliferation status of SOC which has high repeatability and may guide individualized treatment programs.

**Key points:**

• The radiomic–radiological nomogram may guide individualized treatment programs of SOC.

• This radiomic–radiological nomogram showed a favorable prediction ability.

• Homogeneous slightly higher signal intensity on T2FSE is vital for Ki-67.

**Graphical Abstract:**

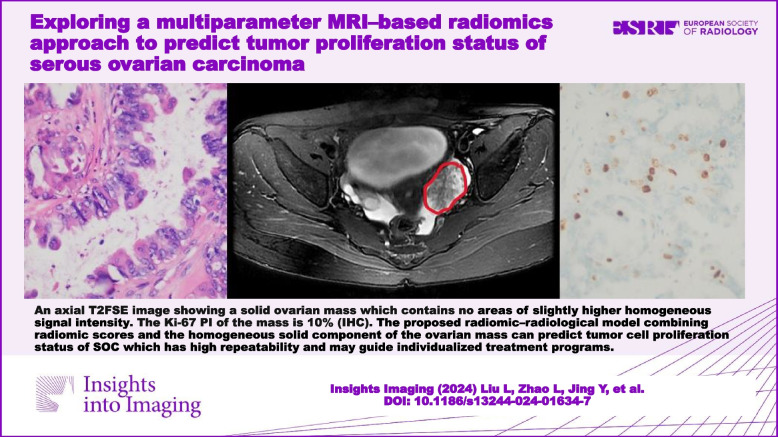

## Introduction

Epithelial ovarian carcinoma accounts for more than 90% ovarian malignancies. Among them, serous ovarian carcinoma (SOC) has the highest incidence and is the most lethal gynecologic malignancy [1–4]. Currently, the standard treatment strategy for SOC is primary debulking surgery (PDS) or neoadjuvant chemotherapy (NACT) followed by interval debulking surgery. Regarding the aggressiveness and clinical behavior of SOC, tumor cell proliferation has important diagnostic and prognostic value. Hence, preoperative evaluation of the tumor proliferation status is important [5–7].

Ki-67 antigen is considered a tumor proliferation status index and has been extensively used both as a diagnostic and prognostic indicator of many malignancies, such as glioma, breast cancer, lung cancer, and liver cancer [8–11]. Ki-67 antigen is a DNA-binding protein that is expressed in all cells during the mitotic cycle. The higher its expression level, the more nuclear fission, indicating more active the cell proliferation. Excessive cell proliferation can easily contain physiological apoptotic DNA or replication error DNA endlessly entering the cell cycle, eventually leading to cell carcinogenesis [12]. Ki-67 antigen is usually overexpressed in malignant ovarian tumor compared with benign or borderline tumors. For ovarian carcinoma, the expression of Ki-67 antigen is widely used to guide clinical management, such as tumor aggression, vascular invasion, tumor metastasis, reserved prognosis, and poor response to chemotherapy [3, 13–16]. At present, Ki-67 expression status is mainly assessed by immunohistochemical (IHC) examination of biopsy or surgery specimens. However, with the IHC sampling method, the evaluation of Ki-67 expression is unrepresentative of its expression level in the entire tumor, which affects clinical decision-making. Therefore, the establishment of models that can predict Ki-67 expression levels of the entire tumor is crucial to guide individualized treatment decision-making and postoperative monitoring of patients with SOC.

SOC usually presents as ovarian cystic, cystic-solid, or solid masses on unilateral or bilateral ovaries on MRI, but how to determine the proliferative status of the masses by imaging methods? Wang et al. [17] used the parameters of apparent diffusion coefficient (ADC) histogram to distinguish the tumor stages of epithelial ovarian cancer and determine the lymph node status and correlations between ADC values and p53 and Ki-67 expressions. However, the histogram is only the extracted first-order features, which are used to describe the intensity distribution of voxels of images but cannot reflect tumor heterogeneity and complexity.

Radiomics, a recently developed technique, can help predict tumor phenotype and heterogeneity and provide information about tumor biological behaviors and pathophysiology based on a large amount of high-throughput data [18, 19]. At present, many researchers have applied radiomics to the diagnosis and differential diagnosis of diseases, efficacy evaluation, prediction, etc. [20–23]. However, too little work has focused on predicting the expression of Ki-67 in patients with SOC based on machine learning or radiomic features extracted from magnetic resonance imaging (MRI).

In this study, we aimed integrated relevant clinical and radiological variables to establish a model based on MRI radiomics to predict tumor cell proliferation status, in which training, internal validation, and external validation sets were designed to test the robustness of the prediction model. If the model proves to be effective, our findings will play a pivotal role in clinical decision-making.

## Materials and methods

### Patients

In total, 3276 patients were initially enrolled in this retrospective study from January 2016 to March 2022 (2779 and 497 patients from institutions A and B, respectively). The two institutional ethics committees approved this study, and the requirement for informed consent was waived owing to the retrospective design.

**Fig. 1:**
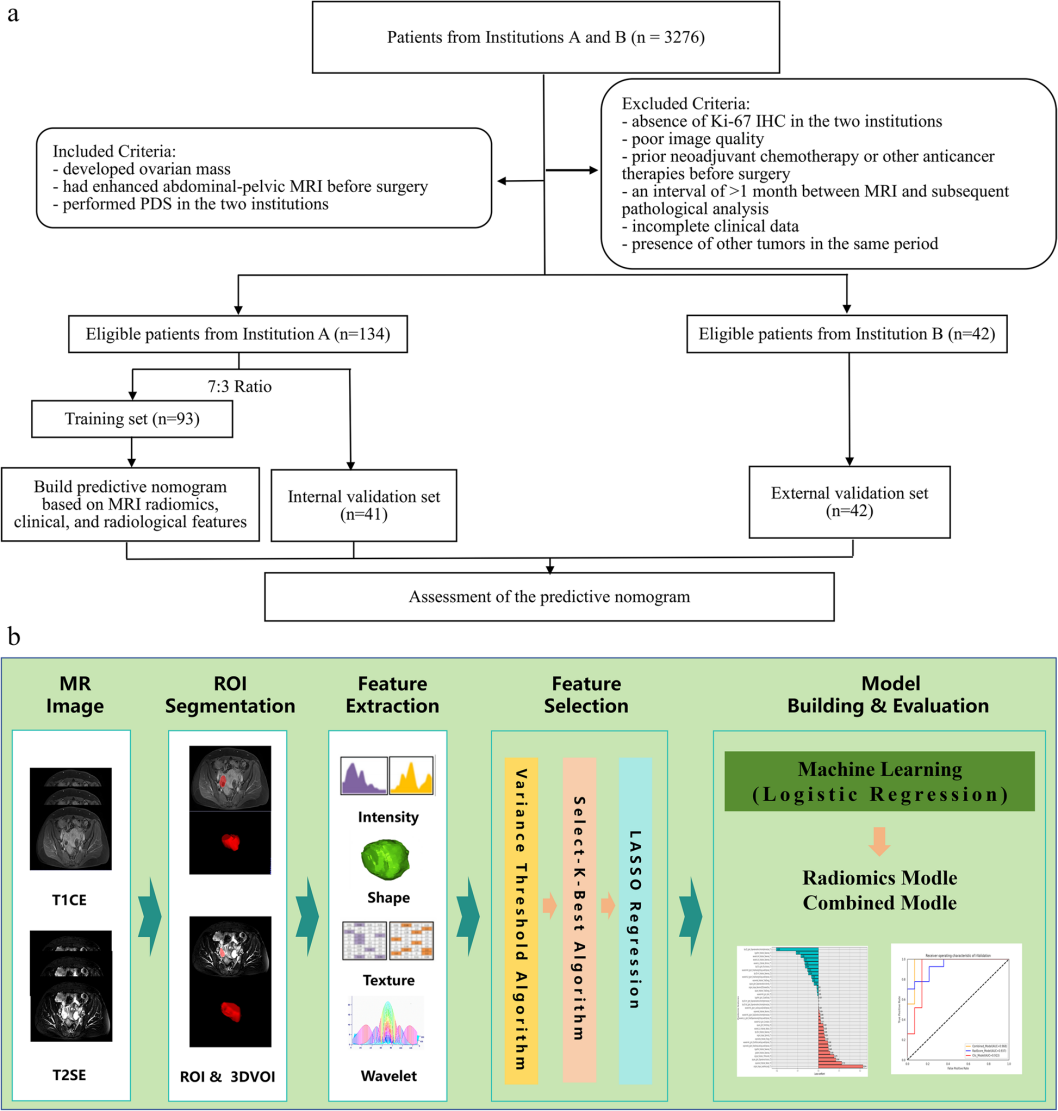
The flow diagram of this study. **a** Workflow of this study. **b** Five steps of our predictive model building: MR image acquisition, ROI segmentation, features extraction, features selection and model building and evaluation. ROI, the region of interest

The inclusion criteria were as follows: (1) developed ovarian mass, (2) underwent PDS in the two institutions, and (3) had enhanced abdominal-pelvic MRI before surgery. The exclusion criteria were as follows: (1) absence of Ki-67 IHC in the two institutions, (2) poor image quality (e.g., artifact), (3) incomplete clinical data, (4) prior NACT, pharmacological treatment or other anticancer therapies before surgery, (5) an interval of > 1 month between MRI and subsequent pathological analysis, and (6) presence of other tumors in the same period. Finally, we screened 134 patients from institution A for the training and internal validation sets, and 42 patients from institution B for the external validation set. A total of 134 patients from institution A were randomly divided into training and internal validation sets, with a ratio of 7:3. The flow diagram of this study is summarized in Fig. 1a.

### MRI acquisition and data collection

All patients from institution A and B underwent abdominal pelvic MRI using 3.0 T (GE) and 1.5 T (GE) MRI systems, respectively. The detailed parameters of the imaging sequences protocol we chose are shown in Table 1. Both T2-weighted imaging fat-suppressed fast spin-echo (T2FSE) and T1 with contrast enhancement (T1CE) are axial, and the contrast medium in both institutions A and B is gadopentetate dimeglumine (0.1 mmol/kg body weight, Magnevist; Bayer Schering).

**Table 1:** The detailed parameters of the imaging sequences

MRI scans	T2FSE					T1CE (LAVA)					
	TR (ms)	TE (ms)	Slice thickness (mm)	Gap (mm)	FOV (cm)	TR (ms)	TE (ms)	Slice thickness (mm)	Gap (mm)	FOV (cm)	Scan time (s)
3.0 T (GE)	4260	108.6	5	5	40	4	2	4	2	40	30, 60, 90, 120
1.5 T (GE)	2700	68	5	5	40	4	2	4	0	40	30, 60, 90, 120

*T2FSE* T2-weighted imaging fat-suppressed fast spin-echo, *T1CE* T1 with contrast enhancement, *LAVA* liver acquisition with volume acceleration 3D with fat saturation, *TR* time of repetition, *TE* time of echo; *FOV*, field of view

Two radiologists with > 10 years of experience in gynecologic MRI were blinded to the clinicopathological information and independently evaluated preoperative MR images of each patient carefully for distribution (unilateral/bilateral), size of the ovarian mass (maximum diameter), shape of the ovarian mass (lobulated/non-lobulated), mass angiogenesis [24] (enlarged blood vessels supplying or draining the tumor observed around or in the center of the ovarian mass), homogeneous solid component of the ovarian mass (solid part containing homogeneous slightly higher signal intensity area on T2FSE), peritoneum/mesentery nodules, metastases of distant parenchymal organs, retroperitoneal lymphadenopathy, and amount of ascites. The spread of ascites in the abdomen and pelvis was quantified as no/small (ascites confined to the pelvic) and medium-to-large (ascites beyond the pelvis). If there was any disagreement, the MR image features were re-evaluated, and a consensus was reached.

The clinical and laboratory data associated with epithelial ovarian carcinoma of all patients were recorded [25], including age, serum carbohydrate antigen 125, human epididymis protein 4, neutrophil-to-lymphocyte ratio, and the International Federation of Gynecology and Obstetrics (FIGO) stage.

### Ki-67 expression assessment

All specimens were fixed in 10% neutral formaldehyde solution, dehydrated conventionally, embedded in paraffin, and cut into sections of 4-μm thick. Hematoxylin and eosin (HE) staining and immunostaining was evaluated qualitatively by two pathologists. The proliferative index (PI) of the lesion was estimated by Ki-67 Mindbomb E3 ubiquitin protein ligase1 (MIB-1) of IHC and was quantitatively expressed as the Ki-67 MIB-1 labeling index when the number of positively stained cells per 100 epithelial cells after counting was at least 1000 in each case by high-power objective of the microscope (× 400) [15, 16]. As there is no international consensus on the cutoff value for percentage of the Ki67 expression, the SOC lesions were divided into two groups according to several studies [15, 16, 26, 27]: the high expression group (PI ≥ 50% immunoreactive cells are positive) and the low expression group (PI < 50% immunoreactive cells are positive).

### Tumor segmentation and feature extraction

The overview of our modeling workflow is shown in Fig. 1b. The pathological changes of the tumor are reflected in T2FSE images, and the blood supply of the tumor is reflected in T1CE images. The region of interest (ROI) was determined by two radiologists (with ≥ 10 years of experience in gynecological MRI) by drawing each ovarian mass boundaries manually slice-by-slice on T2FSE and T1CE images using ITK-SNAP software (version 3.8.0, http://openiconlibrary.sourceforge.net/), which is tumor segmentation. Any discrepancy was resolved by consensus. For patients with bilateral masses, the larger one was selected to delineate the ROI, which usually contained more comprehensive imaging features. Before feature extraction, each MR image was preprocessed as follows: (1) image standardization: a linear interpolation was used to resample the MR images to 1 mm ×1 mm × 5 mm voxel size to ensure image standardization. (2) *Z*-score normalization was conducted to ensure the repeatability of the results and reduce potential effects associated with different sequence parameters and scanner manufacturers [28]. Subsequently, 1688 handcrafted radiomic features from each sequence were extracted using the open-source Python package Pyradiomics (version 3.0.1; http://www.radiomics.io/pyradiomics.html). The extracted radiomic features included four groups: morphologic features, intensity-based (first-order), texture (second-order), and wavelet features formed based on these three feature categories. One month later, we repeatedly delineate the ROI in a subset of the training set with 31 data to assess the repeatability of feature extraction using intraclass correlation coefficients (ICC). In this study, an ICC value > 0.80 was considered robust, and 1686 and 1633 of the initial 1688 image features remained on T2FSE and T1CE sequences, respectively.

### Feature selection, radiomic signature construction, and evaluation

After the repeatability test, three feature selection methods (variance threshold algorithm, SelectKBest algorithm, and least absolute shrinkage and selection operator [LASSO] regression) were used in turn for dimension reduction of each sequence (T2FSE and T1CE) and combined sequences (T1CE–T2FSE). Then, the radiomic scores (Radscores) of each sequence and combined sequences were calculated from the final radiomic features and their respective coefficients. Then, the radiomic features of each sequence (T2FSE and T1CE) and combined sequences (T1CE–T2FSE) were used to establish models to predict Ki-67 expression levels. The receiver operating characteristic curve analysis was used to evaluate the predictive performance, and the DeLong test was performed to compare the differences between them. The optimal radiomic model was the radiomic model with the highest area under the curve (AUC).

### Radiomic–clinical–radiological model construction and evaluation

The best cut-off values for age, CA-125, HE-4, and NLR were determined by univariate regression, respectively, and were converted into categorical variables. Univariate and multivariate analyses were used to obtain the independent predictors of the model among the clinical and radiological variables. Then, the radiomic–clinical–radiological model was obtained by integrating the optimal radiomic scores and the independent predictors using multivariable logistic regression. The predictive performance of the radiomic–clinical–radiological model was assessed using AUC, accuracy, sensitivity, and specificity and validated in the internal or external validation set.

### Statistical analysis

Statistical analyses were performed with the Python package scipy (version 1.7.3, https://scipy.org/), and the radiomic–clinical–radiological model and nomogram were developed using the R software (version 3.5.1, http://www.r-project.org/). Student’s *t*-test and Wilcoxon test were used to assess the differences of continuous variables. Chi-square test was used to assess the differences of categorical variables. The DeLong test was used to assess the differences in the AUC of the models. *p* < 0.05 was defined significant.

## Results

### Features of the study population

A total of 134 patients from institution A were screened based on the inclusion and exclusion criteria, and they were randomly allocated into training (*n* = 93) and internal validation (*n* = 41) sets, with a ratio of 7:3. From institution B, 42 patients were screened for the external validation set. The average age of 134 patients with SOC eligible from institution A was 54 years (range 27–80 years), and that of 42 patients with SOC eligible from institution B was 54 years (range 30–74 years). As shown in Table 2, a significant difference was found only in the amount of ascites among the five clinical and nine radiological variables (*p* < 0.05), indicating that the two data sets were from the same population. After univariate logistic regression, the optimal cutoff values of the continuous variables were as follows: age (cutoff = 55, C-index = 0.652), CA-125 (cutoff = 1231, C-index = 0.559), HE-4 (cutoff = 418, C-index = 0.561), NLR (cutoff = 3.73, C-index = 0.614), and maximum diameter of the ovarian neoplasm (cutoff = 7.4, C-index = 0.646).

**Table 2:** Equilibrium comparison between the training and internal validation sets, and the clinical/radiological characteristics in the external validation

Characteristics	Equilibrium comparison			External validation set, *n* = 42
	Training set, *n* = 93	Internal validation set, *n* = 41	*p* value	
Clinical characteristics				
Age	53.05 ± 10.83	54.24 ± 10.40	0.554^a^	54.31 ± 8.30
FIGO			0.098^c^	
I–II	16 (17.2)	15 (36.6)		7 (16.7)
III–IV	77 (82.8)	26 (63.4)		35 (83.4)
CA125	830.50 (307.70, 1856.10)	593.00 (239.50, 2036.70)	0.327^b^	1000.50 (410.24, 2104.28)
HE4	396.00 (153.00, 767.00)	224.00 (108.00, 597.00)	0.084^b^	572.55 (201.58, 960.12)
NLR	2.87 (1.89, 5.43)	3.26 (2.04, 4.58)	0.186^b^	3.49 (2.57, 5.36)
Radiological characteristics				
Distribution			0.106^c^	
Unilateral	41 (44.1)	25 (61.0)		22 (52.4)
Bilateral	52 (55.9)	16 (39.0)		20 (47.6)
Lobulated ovarian mass			0.547^c^	
No	3 (3.2)	3 (7.3)		3 (7.1)
Yes	90 (96.8)	38 (92.7)		39(92.9)
Angiogenesis of ovarian mass			0.741^c^	
No	8 (8.6)	5 (12.2)		4 (9.5)
Yes	85 (91.4)	36 (87.8)		38 (90.5)
Size of ovarian mass (maximum diameter, cm)	8.20 (6.00, 12.00)	8.60 (6.80, 11.00)	0.835^b^	6.20 (4.82, 8.90)
Homogeneous solid in ovarian mass			1^c^	
No	26 (28.0)	12 (29.3)		12 (28.6)
Yes	67 (72.0)	29 (70.7)		30 (71.4)
Peritoneum/mesentery nodules			0.837^c^	
No	24 (25.8)	12 (29.3)		9 (21.4)
Yes	69 (74.2)	29 (70.7)		33 (78.6)
Metastases of distant parenchymal organs			1^c^	
No	79 (84.9)	35 (85.4)		35 (83.3)
Yes	14 (15.1)	6 (14.6)		7 (16.7)
Retro-peritoneal lymphadenopathy			0.106^c^	
No	73 (78.5)	26 (63.4)		35 (83.3)
Yes	20 (21.5)	15 (36.6)		7 (16.7)
Amount of ascites			0.013^c^	
No/small	38 (40.9)	27 (65.9)		20 (47.6)
Medium-to-large	55 (59.1)	14 (34.1)		22 (52.4)

*FIGO* the International Federation of Gynecology and Obstetrics, *CA125* carbohydrate antigen 125, *HE4* human epididymis protein 4, *NLR* neutrophil-to-lymphocyte ratio; Age is presented as means ± standard deviations; CA-125, HE-4, NLR and Size of ovarian mass are expressed as medians (interquartile ranges); other characteristics are expressed as absolute numbers and percentages

^a^Student’s *t* test

^b^ Wilcoxon test

^c^Chi-square test

Among 134 cases of SOC from hospital A, there were 89 cases of high Ki-67 expression (PI ≥ 50%), accounting for 66.4%, whereas there were 45 cases of low Ki-67 expression (PI < 50%), accounting for 33.6%. And among 42 cases of SOC from hospital B, there were 31 cases of high Ki-67 expression, accounting for 73.8%, whereas there were 11 cases of low expression, accounting for 26.2%.

In the training set, significant differences in variables of the homogeneous solid component of the ovarian mass and FIGO stage were found between the high and low Ki-67 expression groups on the univariate regression analysis (*p <* 0.05), whereas there was no significant difference in clinical variables between the high and low Ki-67 expression groups. On the multivariate regression analysis, only the homogeneous solid component of the ovarian mass was considered the independent predictor of the expression level of Ki-67 (Tables 3 and 4).

**Table 3:** Univariate and multivariate regression analysis of the clinical features

Characteristics	Univariate regression analysis			Multivariate regression analysis		
	OR	95% CI	*p* value	OR	95% CI	*p* value
Age						
≤ 55	1					
> 55	0.987	0.962–1.044	0.924	N/A	N/A	N/A
FIGO						
I – II	1			1		
III–IV	2.08	1.061–4.395	0.040	1.64	0.68–3.99	0.27
CA-125						
≤ 1231	1					
> 1231	0.497	0.19–1.22	0.137	N/A	N/A	N/A
HE4						
≤ 418	1					
> 418	0.483	0.194–1.116	0.109	N/A	N/A	N/A
NLR						
≤ 3.73	1					
>3.73	0.513	0.968–1.643	0.111	N/A	N/A	N/A

Chi-square test *FIGO* the International Federation of Gynecology and Obstetrics, *CA-125* carbohydrate antigen 125, *HE4* human epididymis protein 4, *NLR* neutrophil-to-lymphocyte ratio, *OR* odds ratio, *95% CI* 95% confidence interval

**Table 4:** Univariate and multivariate regression analysis of the radiological features

Characteristics	Univariate regression analysis			Multivariate regression analysis		
	OR	95% CI	*p* Value	OR	95% CI	*p* Value
Distribution						
Unilateral	1					
Bilateral	0.719	0.293–1.717	0.461	N/A	N/A	N/A
Lobulated ovarian mass						
NO	1					
Yes	0.967	0.045–10.845	1	N/A	N/A	N/A
Angiogenesis of ovarian mass						
No	1					
Yes	1.22	0.237–5.344	0.794	N/A	N/A	N/A
Size of ovarian mass (cm)						
≤ 7.4	1					
> 7.4	0.906	0.802–1.019	0.104	N/A	N/A	N/A
Homogeneous solid in ovarian mass						
No	1			1		
Yes	23.94	7.859–86.448	< 0.01	22.15	6.7–73.21	< 0.01
Peritoneum/mesentery nodules						
No	1					
Yes	2.07	0.790–5.430	0.135	N/A	N/A	N/A
Metastases of distant parenchymal organs						
No	1					
Yes	2.01	0.572–9.443	0.312	N/A	N/A	N/A
Retro-peritoneal lymphadenopathy						
No	1					
Yes	1.66	0.570–5.569	0.375	N/A	N/A	N/A
Amount of ascites						
None/ small	1					
Middle/large	1.30	0.541–3.129	0.551	N/A	N/A	N/A

Chi-square test *FIGO* the International Federation of Gynecology and Obstetrics, *CA-125* carbohydrate antigen 125, *HE4* human epididymis protein 4, *NLR* neutrophil-to-lymphocyte ratio, *OR* odds ratio, *95% CI* 95% confidence interval

### Construction and internal and external validation of the radiomic signature

**Fig. 2:**
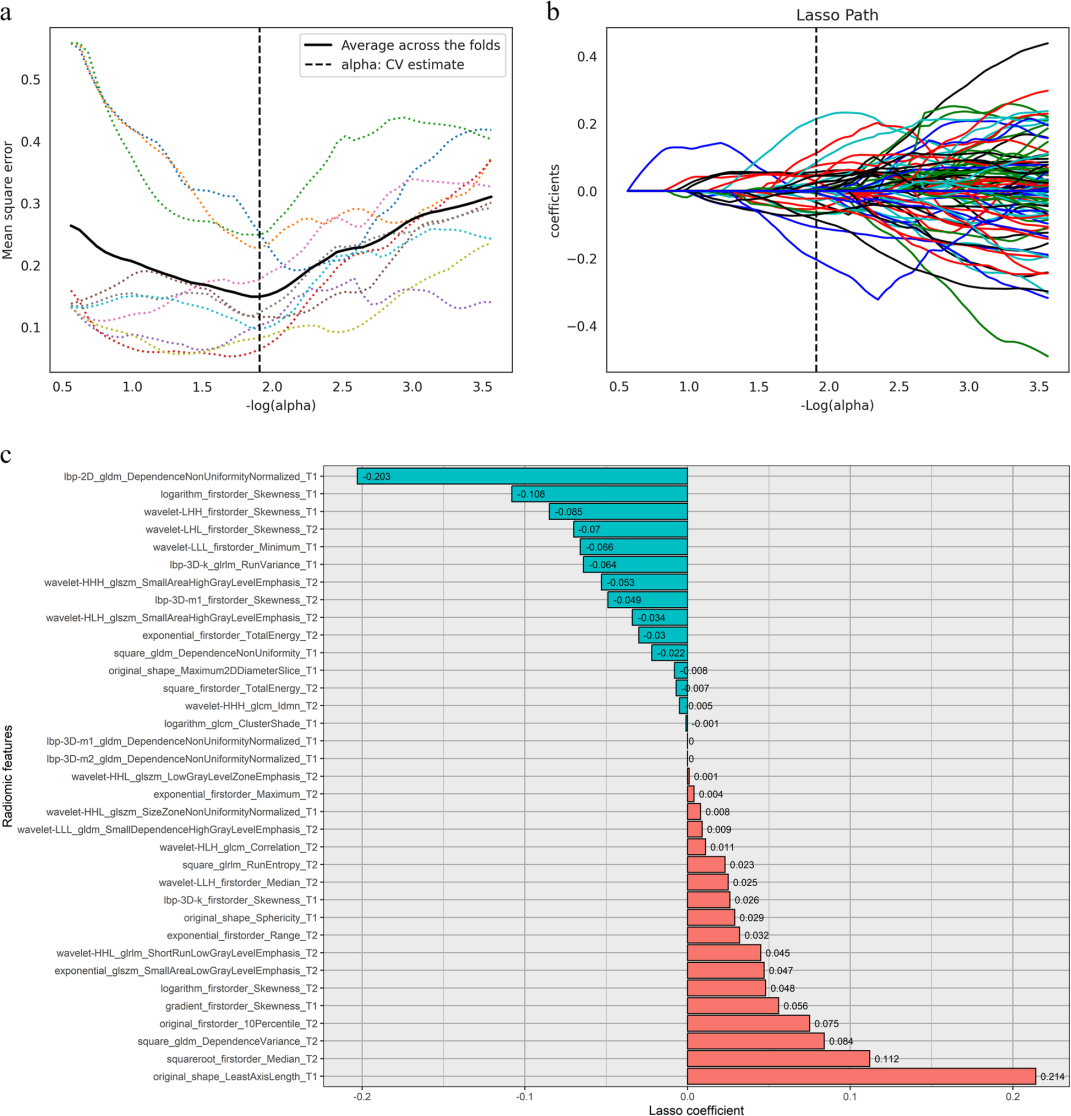
Features retained after radiomics high-throughput feature dimension reduction. **a** MSE PATH, the dotted line represents the *α* value (1.9) with the smallest mean square error. **b** LASSO PATH, the radiomic features are determined according to the *α* value with the smallest mean square error. **c** Lasso coefficient of the features after radiomics high-throughput feature dimension reduction

Extracted from T2FSE and T1CE sequences, we included 1688 radiomic features, both. After the elimination of redundancy using the variance threshold algorithm, SelectKBest algorithm, and LASSO, 20, 15, and 35 features were left from the T2FSE, T1CE, and T1CE–T2FSE, respectively (Fig. 2). Subsequently, the radiomic signature of T2FSE, T1CE, and T1CE–T2FSE was constructed respectively. Compared with the AUC values of each radiomic signature, the AUC value of the radiomic signature (T1CE–T2FSE) was the highest in the training set (0.999 vs. 0.965 and 0.860). The DeLong test showed that the AUC value of the radiomic signature (T1CE–T2FSE) was significantly different from those of the other two radiomic signatures (*p <* 0.05). As a result, the radiomic signature (T1CE–T2FSE) was considered the optimal radiomic signature.

### Establishment and internal and external validations of the radiomic–radiological model

**Fig. 3:**
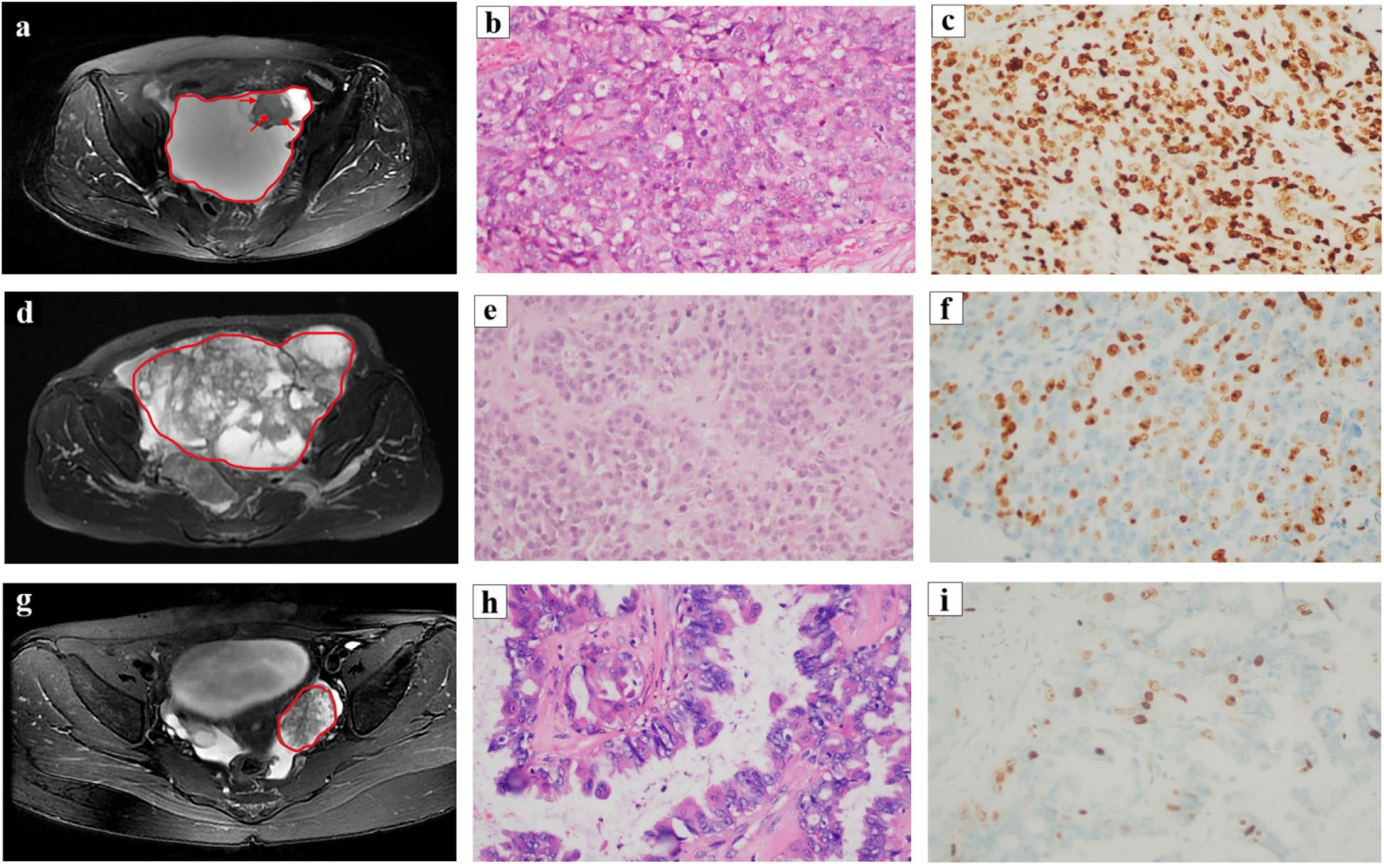
The radiological features on MR images and corresponding HE and Ki-67 of IHC. The radiological features on MR images and corresponding HE (hematoxylin-eosin staining, × 400) and Ki-67 of IHC (Immunohistochemistry], × 400). **a**–**c** A 70-year-old woman with high-grade serous papillary cystadenocarcinoma. **a** An axial T2FSE (T2-weighted imaging fat-suppressed fast spin-echo) image shows a cystic mass with solid nodules in the left ovary (red line) which contains homogeneous slightly higher signal intensity area (red arrow). **b** HE of high-grade serous papillary cystadenocarcinoma. **c** IHC of high-grade serous papillary cystadenocarcinoma displaying high Ki-67 PI (proliferative index) (80%). **d**–**f** A 64-year-old woman with high-grade serous cystadenocarcinoma. **d** An axial T2FSE image shows a fused cystic-solid mass (red line) from bilateral ovaries which looks like sponge and contains no homogeneous slightly higher signal intensity area. **e** HE of high-grade serous cystadenocarcinoma. **f** IHC of high-grade serous cystadenocarcinoma displaying low Ki-67 PI (40%). **g**–**i** A 32-year-old woman with non-invasive micropapillary serous carcinoma. **g** An axial T2FSE image shows a solid mass in the left ovary (red line) which contains no homogeneous slightly higher signal intensity area. **h** HE of non-invasive micropapillary serous carcinoma. **i** IHC of non-invasive micropapillary serous carcinoma low Ki-67 PI (10%)

**Fig. 4:**
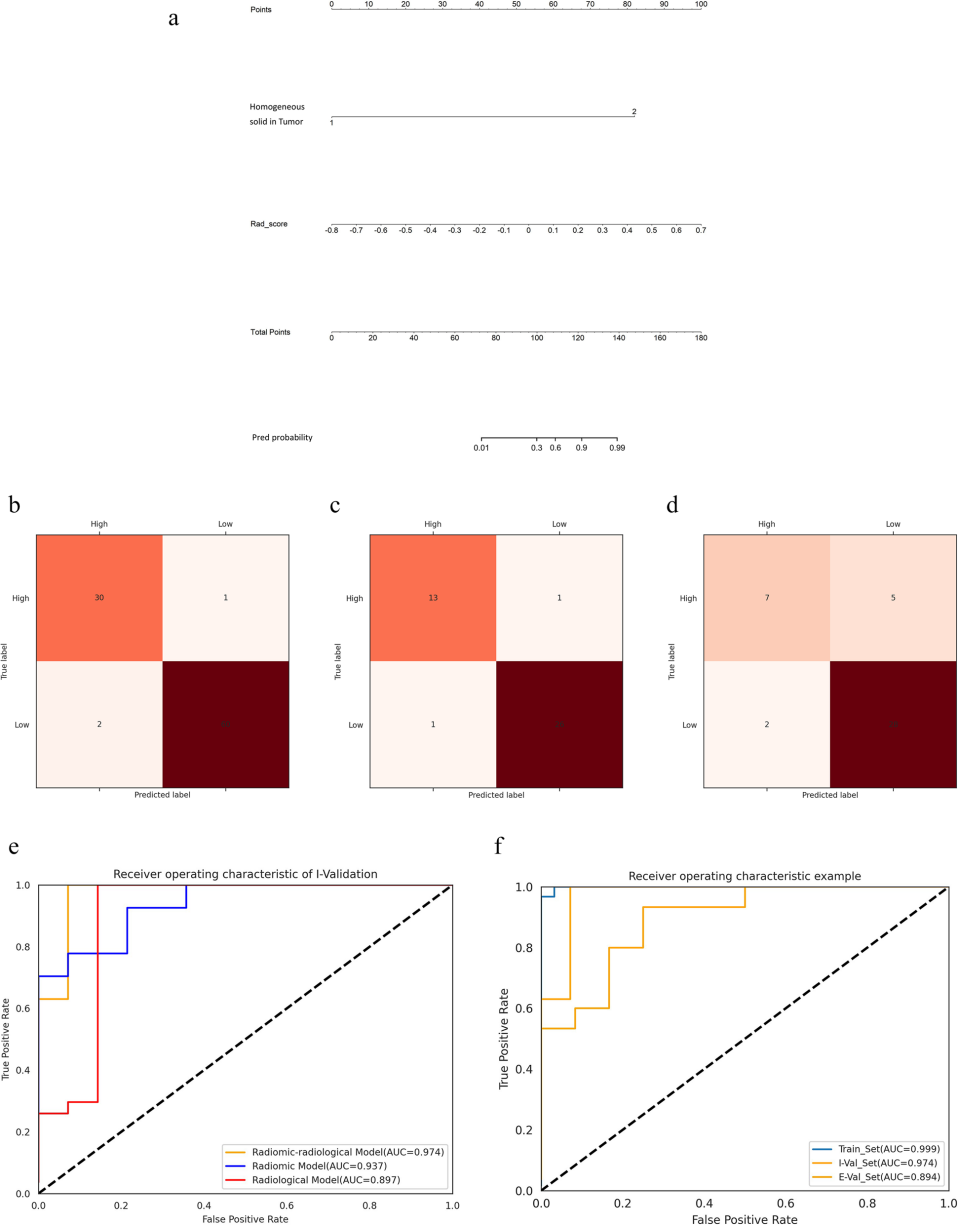
The radiomic-radiological model and its performance. **a** The radiomic-radiological model for the training set. **b**–**d** The confusion matrix of the training, internal validation, and external validation sets, respectively. **e** The ROC curves of the radiomic, radiological, and radiomic-radiological models for the internal validation set. **f** The ROC curves of radiomic-clinical-radiological model for the three sets. ROC, receiver operating characteristic

**Fig. 5:**
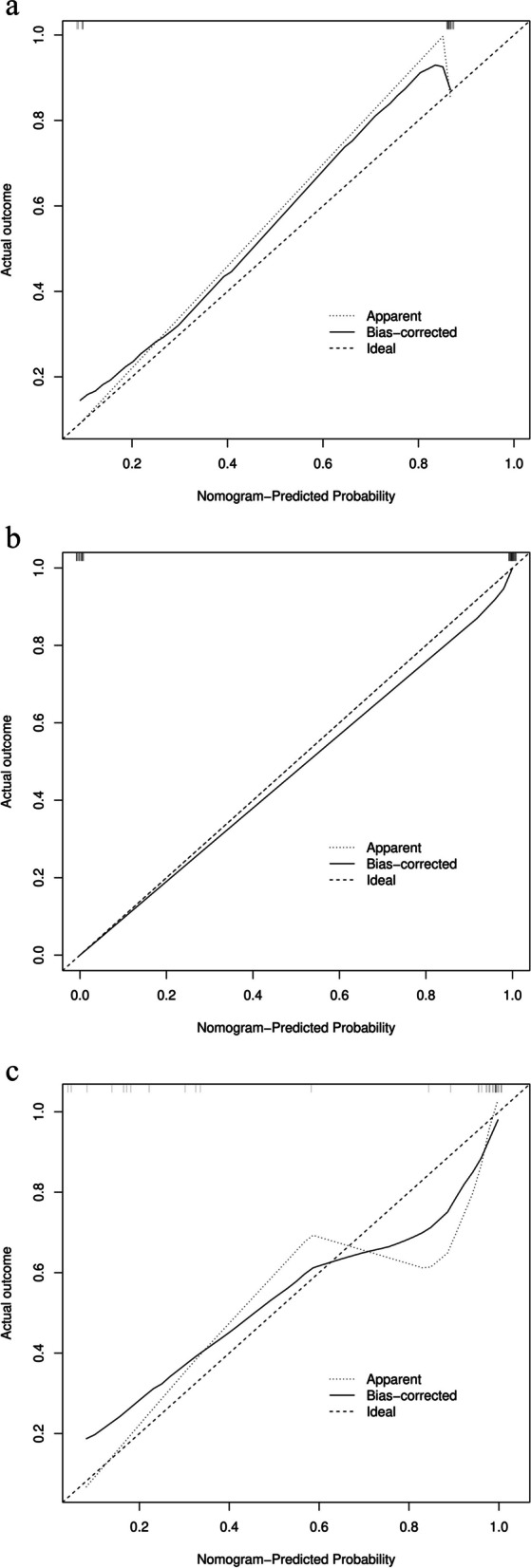
Calibration curves of the radiomic-radiological model in the training, internal validation, and external validation sets. **a** Training set. **b** Internal validation set. **c** External validation set

By univariate and multivariate analyses, only the homogeneous solid component of the ovarian mass was considered the independent predictor (Fig. 3) among the five clinical and nine radiological variables. Thus, we developed a radiological model instead of a clinical–radiological model using the only independent predictor. Likewise, we integrated the optimal radiomic scores and the only independent predictor to establish a radiomic–radiological model instead of radiomic–clinical–radiological model (Fig. 4). Compared with the AUC values of the radiomic signature, radiological model, and radiomic–radiological model, the radiomic–radiological model had the best AUC of the training set of 0.999, internal validation set of 0.974, and external validation set of 0.894. The DeLong test showed a significant difference between the radiological model and the radiomic–radiological model and between the radiomic signature and the radiological model (*p <* 0.05). Moreover, no significant difference was found between the radiomic signature and the radiomic–radiological model (*p* = 0.480) in the training set, whereas the difference was significant in the external validation set (*p* < 0.05). Figure 5 shows the calibration curves of the radiomic–radiological model in the three sets.

## Discussion

In this study, we developed a radiomic–radiological model to predict the Ki-67 expression level of the tumor by extracting features from preoperative or pretreatment MRI of patients with SOC, integrating clinical and radiological variables probably related to tumor proliferation, which was verified in the external validation set. This model demonstrated excellent performance in predicting Ki-67 expression. To our knowledge, this is the first study to establish a radiomic–radiological model to predict the Ki-67 expression levels of SOC using MRI data. The robustness and generalizability of the model were further verified by external validation of the calibration curve.

Before feature extraction, we conducted standardized preprocessing of image data (including voxel size resampling and *Z*-score normalization) according to previous research methods [28] to significantly reduce the differences among scanners and imaging parameters. Wang et al. [17] used the ADC histogram parameters to distinguish different tumor stages of epithelial ovarian cancer and determine lymph node status and correlations between ADC values and Ki-67 expression. Li et al. [29] used ADC histogram parameters to differentiate high-grade SOC from low-grade SOC and correlate those parameters with the Ki-67 proliferation index. These studies indicate that we can use first-order statistics to study the correlation between tumors and IHC indicators of SOC. However, first-order statistics usually describe the voxel intensity distribution of images. Relative to the first- and second-order statistics (texture analysis), radiomics is a high-order statistic using image filter feature transform to reduce noise and enhance edge detection. It can help reveal tumor characteristics that cannot be observed in the original image by naked eyes [30] and play an important role in predicting pathological status [31, 32]. In this study, the radiomic model was established using the 35 most discriminative features extracted (including 3 morphologic, 15 intensity-based [first-order], and 17 texture [second-order] features), on the basis of which 12 wavelet features were formed (Fig. 2).

In our study, the radiomic signature of T1CE–T2FSE had the highest AUC value among the three radiomic signatures (0.999 vs. 0.965 and 0.860). It indicated that multiple sequences can provide more important information than a single sequence, and the performance of the combined model of multiple sequences is higher [33–36]. Although no literature focused on predicting Ki-67 expression of SOC based on radiomics, the result of our study was consistent with that of using radiomics to predict Ki67 of hepatocellular carcinoma [37] and lung cancer [38].

During the study, if the SOC masses were classified according to the traditional method (cystic, cystic-solid, and solid components), the Ki-67 expression of cystic masses might be high (Fig. 3a–c), whereas that of solid masses might be low (Fig. 3d–i). We hypothesized that the expression level of Ki-67 might not significantly correlate with the proportion of the cystic/solid component of the mass. After repeated careful observations, we found that some of the solid masses with low Ki-67 expression were either polycystic or spongiform (Fig. 3d–f), and they did not contain areas of slightly hyperintense T2FSE homogeneity. In our study cohort, we found that the expression level of Ki-67 was mostly higher if the solid part of the ovarian mass contained a homogeneous slightly hyperintense T2FSE area, and lower if not. As a result, we tried to divide the characteristics of the ovarian masses into a new category according to the degree of signal uniformity in the solid component: including homogeneous slightly higher signal intensity area or not on T2FSE. This is a new classification of the ovarian mass, which is independent of the presence or absence of a cystic component of the mass or the percentage of cystic/solid component of the mass. In our study, the ovarian mass has higher expression levels of Ki-67 if the solid part contains homogeneous slightly higher signal intensity area on T2FSE. By univariate and multivariate analyses, a significant difference in this mass feature was found between the high and low Ki-67 expression groups compared with other variables. In other words, homogeneous slightly higher signal intensity area on T2FSE indicates the presence of densely packed cells in the mass, which may be related to the higher degree of tumor proliferation.

In addition to radiomic signature, we included a total of five clinical and nine radiological factors that may have significance for tumor proliferation status, including FIGO stage, serum tumor markers, tumor maximum diameter, homogeneous solid component of the ovarian mass, lobulation, tumor angiogenesis, and peritoneal metastases. However, by univariate and multivariate analyses, only the homogeneous solid component of the ovarian mass was confirmed to be the independent predictor. According to our analysis, because SOC is highly malignant and often has a large volume when it is found, there is a large amount of ascites with peritoneal metastasis. The lobulation sign and tumor angiogenesis are also malignant characteristics of ovarian tumors. However, the cases in our study were all SOC. Therefore, this may be the reason why there were no significant differences between the high and low expression groups of Ki-67 among other clinical and radiological characteristics in this study.

Wang et al. [17] demonstrated there was a negative correlation between apparent diffusion coefficient parameters and Ki-67 labeling index values using ADC histogram analysis. To develop a nomogram predicting the expression of Ki-67, which is more intuitive compared to the result of Wang et al. [17], we integrated the optimal radiomic scores and the radiological independent predictor determined by univariate and multivariate analyses. The AUC of the radiomic–radiological model is significantly higher than that of the optimal radiomic signature or the radiological model in the internal validation set (0.974 vs. 0.937 and 0.897) and external validation set (0.894 vs. 0.725 and 0.867). The AUC of our radiomic–radiological model was significantly higher than that of ADC histogram analysis by Li et al. [29] (0.717–0.807).

This study has several limitations. First, a potential selection bias exists in terms of the inclusion of patients owing to the retrospective design. Second, the sample size can still be considered relatively small for the development of prognostic models. Third, although some studies support the use of 50% as the cut-off value [15, 16, 26, 27], there is no clear consensus on the Ki-67 expression level in SOC. Fourth, through the DeLong test, we found no significant difference between the performance of the radiomic–radiological and the radiomic models in the training set, whereas a significant difference was found in the external validation set. According to our analysis, the AUC of both models in the training set was 0.999; thus, the lack of a significant statistical difference between them was reasonable. Such a result may be related to the selection of our samples (all of them SOC) or sample size; however, it at least indicates that the radiomic model and radiomic–radiological model both have an excellent ability to identify the high and low expressions of ki-67 in SOC. In addition, the difference between the two models was significant in the external validation set, which also suggests a certain generalizability of the model. Fifth, clinical variables were relatively few, such as patient comorbidities, family history, or other molecular biomarkers. Hence, in future studies, we will include multiple pathological subtypes of ovarian carcinoma, expand the sample size, and include more relevant variables for prospective studies and prospectively develop objective assessment criteria for the percentage determination of Ki-67.

## Conclusion

We established a radiomic–radiological model to accurately predict the expression level of Ki-67, which also demonstrated perfect performance in the external validation set. This method has high repeatability and may enable more targeted, effective treatment strategies and clinical monitoring of patients with SOC.

## Data Availability

The datasets used and/or analyzed during the current study are available from the corresponding author on reasonable request.

## Abbreviations

AUC: The area under the receiver operating characteristic curve
CA-125: Carbohydrate antigen 125
FIGO: International Federation of Gynecology and Obstetrics
HE-4: Human epididymis protein 4
NLR: Neutrophil-to-lymphocyte ratio
PDS: Primary debulking surgery
ROC: Receiver operating characteristic
SOC: Serous ovarian carcinoma
